# Utilizing a Suction Catheter as a Makeshift Tracheostomy Tube in a Premature Infant: A Case Report

**DOI:** 10.1155/crot/5109316

**Published:** 2026-02-16

**Authors:** Ashwini V. Bandi, Shaina W. Gong, Gabriela G. Cruz, Alicia S. Lore, Kunal Shetty, Sancak Yuksel

**Affiliations:** ^1^ McGovern Medical School at the University of Texas Health Science Center at Houston, Houston, Texas, USA, uth.edu; ^2^ Department of Otorhinolaryngology-Head and Neck Surgery, The University of Texas Health Science Center at Houston, Houston, Texas, USA, uth.edu

**Keywords:** congenital airway obstruction, custom tracheostomy, pediatric airway, pediatric tracheostomy, suction catheter

## Abstract

One of the most challenging aspects regarding pediatric tracheostomy tube placement is the size selection, particularly in preterm infants. We present the first reported case of a makeshift tracheostomy tube using a 6‐French suction catheter in a 31‐week‐old premature infant in an emergent situation. This approach holds promise as a potentially life‐saving intervention for extremely small preterm infants. Furthermore, this case emphasizes the size discrepancy between the premature neonatal airway and the smallest tracheostomy tubes available. Further research is warranted to allow for more diverse clinical solutions that can accommodate similar cases in acute settings.

## 1. Introduction

A tracheostomy, which consists of a surgically created stoma that provides an alternative airway, can be a life‐saving airway management procedure. Its use was common within the pediatric population as early as the mid‐19^th^ century when it was largely utilized for the management of upper airway obstruction (UAO) caused by acute infectious diseases. The introduction of vaccinations significantly reduced its practice beginning in the 1980s, and today its use within the pediatric population is vastly different [1, 2].

Currently, the most common indications for pediatric tracheostomies are for the management of UAO involving structural airway abnormalities due to both acquired and congenital etiologies. Furthermore, tracheostomies are created for the purpose of long‐term mechanical ventilation secondary to chronic respiratory failure [3, 4]. Within the United States specifically, tracheostomies are performed with increasing rates for the management of disorders related to preterm birth or low birthweight [5, 6].

Within the pediatric population, risks for complications are inversely associated with patient age [7]. Notably, one of the most challenging aspects regarding pediatric tracheostomy is the size selection of the tracheostomy tube. Choosing a tracheostomy tube that is too large for a patient can cause damage to the trachea or larynx, potentially leading to secondary issues like vocal cord granulomas, ulcerations, laryngeal web formation, and subglottic or tracheal stenosis [3]. To avoid such adverse complications, an age‐based formula is typically utilized to help guide size selection of the tracheostomy tube. Unfortunately, the formula has reported inaccuracy rates as high as 60%. Moreover, even the currently established height‐based formulas are unreliable, as height measurements are often difficult to attain in certain situations [8, 9].

We present the first reported case of an emergent tracheostomy using a 6‐French (Fr) suction catheter as a makeshift tracheostomy tube in a 31‐week‐old premature neonate.

## 2. Case Presentation

The patient was born prematurely at 31 weeks gestational age (GA) due to intrauterine growth restrictions, with a weight of 1180 g (12th percentile) and a length of 38 cm (15th percentile). Immediately after birth, the infant experienced respiratory distress and bradycardia which led to multiple rounds of positive pressure ventilation (PPV) and an oral intubation attempt at 6 min of life (MOL). The patient vocalized loudly after intubation, which raised concern for incorrect placement of the endotracheal tube (ETT). Upon evaluation by the pediatric anesthesia team, the ETT was observed to be incorrectly placed in the esophageal inlet. The anesthesia team then attempted to intubate with a 3.0 and a 2.5 uncuffed ETT without success, with both attempts failing to pass the vocal cords due to the infant’s small airway. The patient was placed on nasal continuous positive airway pressure (CPAP), and the otolaryngology team was urgently called to assist with the airway. Due to visible subcostal retractions, a decision was made to emergently transfer the patient to the operating room for further airway evaluation.

With the patient under anesthesia, a straight Miller blade allowed a grade 1 visualization of the larynx. Three attempts to intubate were made. Initially, a rigid bronchoscope was inserted but was unable to pass the subglottis. Then, a neonatal‐sized operating scope was inserted but failed to pass due to a complete tracheal ring observed below the subglottis. Finally, a 2.0 uncuffed ETT was inserted but similarly failed to pass the complete tracheal ring. When the patient began to desaturate to an SpO_2_ of 70%, the instruments were removed, and bag mask ventilation was initiated. There was no prolonged hypoxia.

**FIGURE 1:**
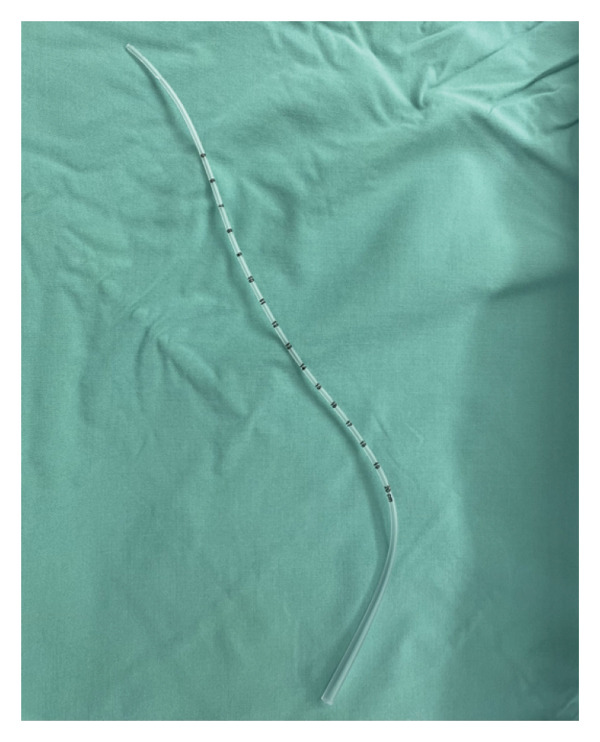
6‐French suction catheter.

After the failed intubation attempts, the surgical team chose to convert the procedure to an emergent open tracheostomy. Once the tracheostomy was created, insertion of a neonatal 2.5 uncuffed tracheostomy tube, the smallest size available, was unsuccessful as it was too large for the patient’s small airway. Insertion of a 2.0 uncuffed ETT was able to pass through the stoma. However, the tight fit of the tube caused intermittent loss of end‐tidal carbon dioxide (CO_2_). The option of extracorporeal membrane oxygenation (ECMO) support was discussed, but given the infant’s 1.18 kg weight, he was not a candidate. A custom tracheostomy tube was created using an 8‐Fr flexible suction catheter and successfully inserted into the airway with some resistance. Notably, the patient ventilated well with this intervention. The 8‐Fr catheter was removed and replaced with a 6‐Fr suction catheter, which allowed for an easier fit into the distal airway without resistance (Figure 1). End‐tidal CO_2_ and adequate ventilation were consistently obtained through the rest of the case.

**FIGURE 2:**
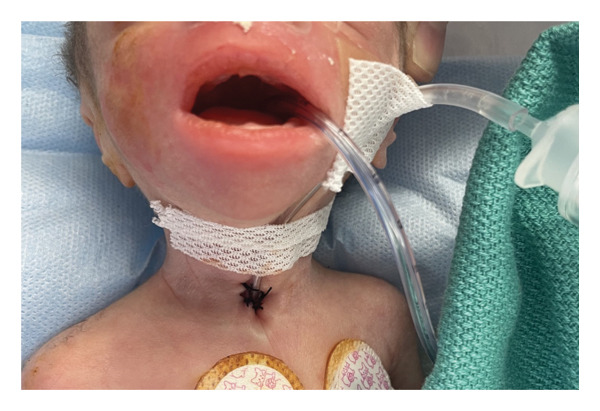
Suction catheter secured to patient’s neck and cheek.

To determine the length to cut the suction catheter, the smallest operating telescope available was inserted into the stoma to identify the carina, which measured 2.5 cm away from the tracheal stoma. This measurement was used to calculate the length of the suction catheter needed to be in the patient’s airway, which was roughly 2 cm above the carina. The infant’s makeshift tracheostomy tube was secured with liquid adhesive and taped to the patient’s neck and cheek (Figure 2). After the customized suction catheter was secured in the airway, the trachea was sutured directly to the skin to allow easier tracheostomy exchanges, and the catheter tube was secured to the skin using silk sutures with a mark on the catheter indicating the position on the stoma. With the securing of the airway, the patient was then transferred to the intensive care unit and followed closely.

During the postoperative period, the suction catheter tube was periodically exchanged with a fresh suction catheter to maintain tube patency. Respiratory toilet was maintained by connecting suction catheter tube directly to suction. When the patient was 1 month old, the tracheostomy tube was exchanged to an 8‐Fr suction catheter. At 1.5 months old, weighing 2 kg, the otolaryngology team upsized the tube to a 2.0 cuffless ETT. Later, this was exchanged to a 2.5 cuffed ETT. Finally, at 2 months of age, the patient underwent exchange of the ETT to a neonatal 2.5 uncuffed tracheostomy tube. At 3 months of age, formal direct laryngoscopy and bronchoscopy was performed showing complete tracheal rings with congenital subglottic stenosis. Patient’s tracheostomy tube was later upsized to a neonatal 3.5 uncuffed tracheostomy tube at 5 months of age.

## 3. Discussion

Infant tracheostomies present a unique surgical challenge, especially in preterm low‐weight neonates. The most challenging and limiting aspect of the procedure is the selection of an appropriately sized tracheostomy tube that is small enough to fit into the airway but still able to provide adequate ventilation. This is especially difficult to manage with premature neonates, as the inner diameter of the subglottis can be as small as 2 mm for low‐weight premature neonates. This translates to a size discrepancy between the neonatal airway and the smallest tracheostomy tubes available [10, 11]. This factor considerably contributes to the morbidity involved in infant tracheostomies with potential complications such as tracheal stenosis, false passage creation, tracheocutaneous fistulas, tube plugging, and stomal granulation [12, 13].

In this case report we delineate the difficulties encountered in securing the airway of a 31‐week‐old preterm infant following multiple failed intubation attempts. The smallest available tracheostomy tube, a neonatal 2.5 uncuffed tracheostomy tube with an outer diameter (OD) of 4.0 mm, failed to fit into the infant’s airway. A 2.0 uncuffed ETT with an OD of 2.9 mm was able to fit but was not able to provide stable ventilation. Ultimately, a 6‐Fr suction catheter with an OD of 2.0 mm was secured into the tracheal stoma with tape, serving as a makeshift tracheostomy tube. This afforded the patient time to grow until they were upsized to an 8‐Fr suction catheter (OD 2.67 mm) and eventually a neonatal 2.5 uncuffed tracheostomy tube at 2 months of age.

To our knowledge, this is the first report of a repurposed suction catheter used as a makeshift tracheostomy tube in an infant’s airway. This approach holds promise as a potentially life‐saving intervention for extremely small preterm infants. This case is evidence of the variation in airway size in preterm infants, especially those with congenital airway anomalies, and emphasizes the size discrepancy between the premature neonatal airway and the smallest tracheostomy tubes available. Thus, it is recommended that suitable equipment be made and distributed for future similar cases.

## 4. Conclusion

We present the first emergent tracheostomy of a premature infant with a 6‐Fr suction catheter repurposed as a custom neonatal tracheostomy tube. This approach proved successful in securing the airway of the infant and may serve as a potential life‐saving intervention for extremely small preterm infants. Moreover, this case emphasizes the size discrepancy between the premature neonatal airway and the smallest tracheostomy tubes available. Further research is warranted to allow for more diverse clinical solutions that can accommodate similar cases in acute settings.

## Funding

No funding was received for this manuscript.

## Consent

Written consent was obtained.

## Conflicts of Interest

The authors declare no conflicts of interest.

## Data Availability

The data that support the findings of this study are available on request from the corresponding author. The data are not publicly available due to privacy or ethical restrictions.
